# Missense Constraint in Intrinsically Disordered Proteins Enhances Missense Variant Interpretation in Neurodevelopmental Disorders

**DOI:** 10.3390/genes17020219

**Published:** 2026-02-10

**Authors:** Nazareth D. J. Robles, Silvio C. E. Tosatto, Maria Cristina Aspromonte

**Affiliations:** 1 Department in Biomedical Sciences, University of Padova, 35131 Padova, Italy; nazarethdejesus.roblesazugaray@studenti.unipd.it; 2 Institute of Biomembranes, Bioenergetics and Molecular Biotechnologies, National Research Council (CNR-IBIOM), 70126 Bari, Italy

**Keywords:** Missense Tolerance Ratio (MTR), intrinsically disordered regions (IDRs), intrinsically disordered proteins (IDPs), neurodevelopmental disorders (NDDs)

## Abstract

Background/Objectives: Interpreting missense variants in intrinsically disordered proteins (IDPs) remains a major challenge, as these proteins lack stable structure and are under-represented in experimental and clinical annotations. Variants occurring in IDPs are disproportionately classified as variants of uncertain significance (VUS), reflecting the absence of appropriate predictive tools rather than true biological neutrality. Here, we address this challenge using a curated dataset of neurodevelopmental disorder (NDD)-associated proteins. Methods: We integrated curated and predicted disorder annotations from DisProt and MobiDB to characterize the structural landscape of 339 NDD-associated proteins. To quantify a regional genetic constraint, we recalculated the Missense Tolerance Ratio (MTR) using a published framework adapted to the recent gnomAD release (v4.1.0). Integration with 33,124 ClinVar-reported missense variants revealed that, while mean constraint levels differ only modestly across structural states, ordered and structural transition regions show the strongest depletion of missense variation. Results: MTR identifies localized low-tolerance subregions within IDRs, indicating that these regions are not uniformly permissive and can harbor functionally essential elements. Conclusions: Overall, our results demonstrate that missense constraint in NDD proteins is highly localized and context-dependent, and that integrating high-quality disorder annotations with updated MTR profiles can improve the prioritization and interpretation of missense variants in IDRs and IDPs.

## 1. Introduction

Neurodevelopmental disorders (NDDs) are genetically heterogeneous conditions often caused by rare, deleterious variants in genes critical for brain development and function [1]. Technological advances in genomics have enabled the identification of a huge amount of rare genetic data expanding our understanding of NDD-associated genes and providing a valuable reference for interpreting missense variation in clinically relevant genes [2,3,4]. Analyses of rare genetic variation have identified hundreds of genes associated with NDDs. However, despite the frequent implication of missense variants [5,6,7], their functional consequences remain challenging to interpret because of strong position-specific effects. Large-scale community efforts, such as the Critical Assessment of Genome Interpretation (CAGI), have further highlighted both the progress and the persistent challenges in accurately predicting the functional and clinical impact of rare missense variants in NDDs and other pathological conditions [8,9,10].

NDD-associated genes encode intrinsically disordered proteins (IDPs) [or proteins with intrinsically disordered regions (IDRs)]. IDRs and IDPs lack a stable three-dimensional structure but play central roles in cellular regulatory pathways, including transcription, signaling, cytoskeleton organization, cell cycle control, receptor regulation, biomineralization, and chaperone functions [11,12,13].

Recent impetus of the field is generated by the observation that IDPs associated with NDDs participate in liquid–liquid phase separation (LLPS), a fundamental cellular mechanism in neurons as well as in other cell types [14]. Despite their functional relevance, IDRs remain poorly characterized in variant interpretation frameworks. Most predictors such as SIFT [15], PolyPhen-2 [16] and MutationTaster2 [17], were developed and trained on globular proteins. Previous studies have reported substantial discordance between computational pathogenicity predictions and clinical classifications for variants located in IDRs. In particular, recent deep learning-based models show reduced agreement with ClinVar [18] for variants in IDRs and IDPs, highlighting persistent challenges in variant interpretation in disordered contexts [19,20]. However, these observations have not yet been systematically incorporated into the clinical interpretation of missense variation in NDDs.

Genetic constraint metrics such as the Missense Tolerance Ratio (MTR) provide an opportunity to better capture regional intolerance to variation by comparing observed and expected missense burdens at high resolution [21]. Previous applications of MTR have explored regional intolerance to missense variation across human protein-coding genes using 240,000 exome and genome sequences. Similarly, deep catalogs of genetic variation have enabled the identification of constrained coding regions (CCRs), revealing that these regions are enriched in pathogenic variants reported in ClinVar and mutations causing NDDs. These studies also highlight protein domains under strong selective pressure and suggest unannotated or incomplete functional regions [18,22]. However, the behavior of MTR within IDRs remains underexplored, particularly given the increasing availability of large population datasets such as gnomAD v4.1.0 [23].

In this study, we integrate curated disorder annotations from DisProt [24] and consensus predictions from MobiDB [25] with an updated calculation of MTR score across 339 proteins implicated in NDDs. Our goal is to characterize the landscape of missense constraint across four structural states: ordered, disordered, disorder transition, and missing residue, and to determine whether a local constraint can reveal functionally important elements within IDRs that are overlooked by current annotation and prediction strategies. Furthermore, by mapping ClinVar variants into structural and constraint features, we assess how regional intolerance influences the distribution of pathogenic, benign, and uncertain variants in NDD genes. Overall, this work provides a systematic evaluation of missense constraint in IDRs/IDPs and demonstrates how combining high-quality disorder annotations with residue-level constraint metrics can improve the interpretation of missense variants in IDRs.

## 2. Materials and Methods

**Figure 1:**
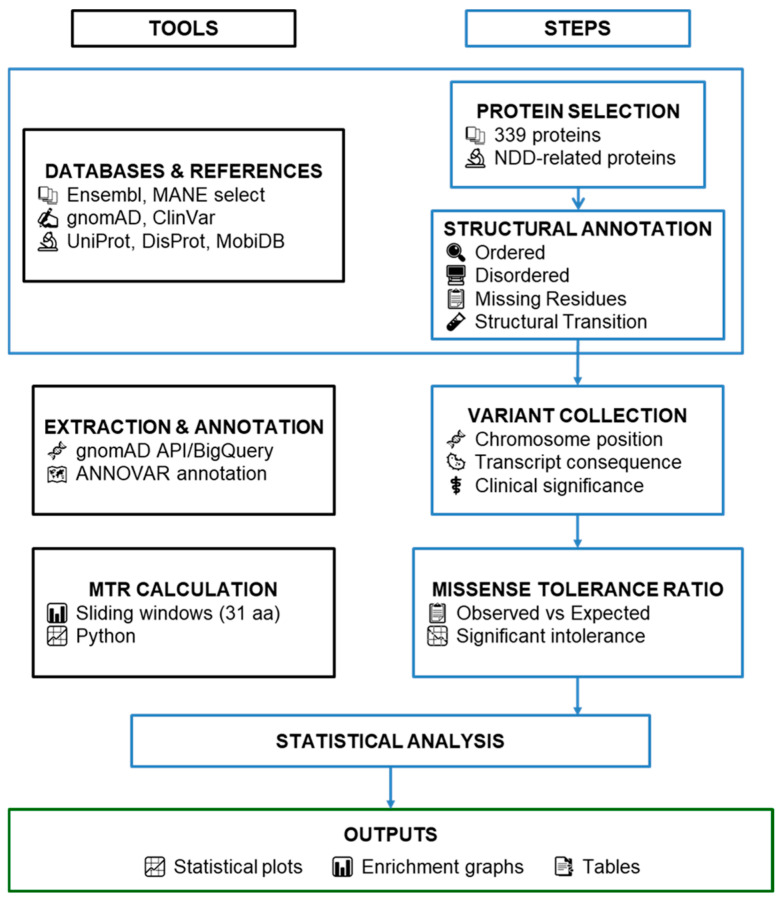
Integrative workflow for variant annotation, structural mapping, and statistical analysis.

We applied a multi-set computational methodology, combining population genetics and protein structural databases. Below we explain in detail each step and we summarize the workflow in Figure 1.

### 2.1. Proteins Selection, Structural Annotation and GO/IDPO Representation

A curated dataset of 339 NDD-related proteins was extracted from the DisProt database, using its “Thematic datasets” that focus on IDPs/IDRs in specific biological and pathological contexts. The NDD proteins list was assembled by integrating specific resources, including SFARI [26], SysNDD (https://sysndd.dbmr.unibe.ch/) and DBD databases (https://dbd.geisingeradmi.org/). Each DisProt entry was cross-referenced with its corresponding UniProt identifier in order to retrieve reviewed, manually annotated protein sequence isoforms [27]. All sequences in the dataset are manually reviewed UniProt entries, ensuring high sequence quality and completeness. Structural disorder annotations were then consolidated using both DisProt and MobiDB databases. From DisProt, we extracted manually curated disorder states, structural transition, and associated functional annotations. From MobiDB, we incorporated consensus disorder predictions and mapped missing residues from Protein Databank (PDB) [28].

The functional Gene Ontology (GO) [29] and Intrinsically Disordered Protein Ontology (IDPO) (https://idpcentral.org/ontology/idpo/, accessed on 1 December 2025 ) representation has been performed using manually curated functions in DisProt across all 339 NDD-related proteins. Terms from the *Biological Process* and *Molecular Function* GO namespaces, as well as from the *Disorder Function* namespace of IDPO, were extracted. The most represented terms are reported together with the number of NDD-associated genes containing at least one DisProt-annotated region assigned to each term, as well as the subset of genes with the highest number of DisProt annotations.

### 2.2. Variants Annotation and Data Mining

The protein list was further refined by aligning the Ensembl MANE select v1.4 set (GRCh38) canonical transcripts [30] using the BioMart server version 0.7 data mining tool (https://www.ensembl.org/info/data/biomart/index.html, accessed on 1 December 2025) provided by Ensembl v115 [31] to ensure the consistency of genomic coordinates in the GRCh38 reference genome (Supplementary Table S1). Transcript discrepancies across gnomAD v4.1.0, Ensembl v115, and MANE select v1.4 set (GRCh38) annotations were manually verified. Single nucleotide missense variants were retrieved via gnomAD v4.1.0 GraphQL API and Big Query pipelines, formatted as Variant Call Format (VCF) files. We relied on the gnomAD v4 release call set and its published QC framework. High-coverage genomic intervals are defined based on per-interval coverage across samples, and sample-level QC includes multiple hard filters and contamination-aware procedures. We included only variants annotated with FILTER = PASS, corresponding to release-quality variants that meet all the gnomAD QC criteria. No additional variant-level QC filters beyond restricting to SNVs were applied in our study. The resulting variant information was subsequently annotated using ANNOVAR (build date 2 March 2025; verified via ANNOVAR web-based update check) [32], on the hg38 reference genome. Annotations included ref Gene With Ver for gene models and ClinVar (2024) for clinical significance, yielding a master file with variant details, predicted consequences, amino acid changes, coverage metrics, and pathogenicity classifications. This integration allows us to continue with the downstream variant analysis.

### 2.3. Calculation of Missense Tolerance Ratio (MTR)

The Missense Tolerance Ratio (MTR) was calculated following the general framework described by Silk et al. (2019) [21], with an important modification: the analysis was performed using the most recent gnomAD release (v4.1.0), which includes 730,947 exomes and 76,215 genomes, substantially expanding the available population variation compared to earlier versions. Single Nucleotide Variants (SNVs), synonymous and non-synonymous (or missense) were mapped along their corresponding canonical protein sequence, assigning to each amino acid position the variants falling within a sliding window of 31 residues, shifted by one nucleotide at a time across the transcript. For each window, we computed the *observed* and *expected* proportions of SNVs. The observed proportion was derived from the number of synonymous and missense variants present in gnomAD v4.1.0 and reported in our VCF files, calculated as

(1) $$Observed\;missense\;proportion=\frac{Observed\;missense\;variants}{\left(Observed\;missense\;variants+Observed\;synonymous\;variants\right)}$$

The expected number of variants was obtained from enumerating all possible SNVs in the transcript, then translated using the genetic code and classified as either missense or synonymous. The expected proportion was calculated as

(2) $$Expected\;missense\;proportion=\frac{Expected\;missense\;variants}{\left(Expected\;missense\;variants+Expected\;synonymous\;variants\right)}$$

With both proportions, the MTR score per residue was computed as

$$MTR=\frac{Observed\;missense\;proportion}{Expected\;missense\;proportion}$$

with lower scores indicating stronger selective constraints. Significantly intolerant regions were identified via FDR-adjusted exact binomial tests comparing observed to expected missense proportions.

### 2.4. Residue-Level Constraint, Composition, and Patterning Analyses

Downstream analyses were performed on a residue-level dataset derived from the 339 NDD-related proteins, combining the canonical UniProt sequence, structural state annotations, and per-residue MTR score. The mutational constraint was assessed using the MTR score as a proxy for intolerance to missense variation. Residues were classified as Intolerant (MTR < 0.4) and Tolerant (MTR ≥ 0.4). The MTR threshold was selected to capture strongly constrained residues in NDD-associated proteins, maintaining adequate coverage and statistical power. Previous studies have shown that stringent cutoffs (e.g., MTR < 0.25 or < 0.5) identify residues enriched for pathogenic variation and highlight functionally important regions, including those not conserved across species [21,33].

Within each structural state and constraint class, we computed amino acid frequencies and their differences between Intolerant and Tolerant residues. Moreover, we evaluated biophysical descriptors like mean side-chain charge, mean Kyte–Doolittle hydropathy, and mean aromatic fraction (F/Y/W) and their corresponding intolerance scores.

For the segment-level patterning analysis, residues were grouped into contiguous stretches within each gene and structural state, yielding segments characterized by length and aromatic spacing. These summaries, together with their Intolerant–Tolerant differences, were used to construct amino acid composition and frequency, the biophysical property and continuous segment patterning heatmaps.

### 2.5. Clinically Significant Variant Enrichment

Variants annotated in the master variant file were extracted based on the ClinVar clinical classification: pathogenic, benign and variant of uncertain significance (VUS). These variants were subsequently mapped onto the MTR values for each protein to investigate correlations between variant pathogenicity and regional genetic constraint, as indicated by MTR scores.

### 2.6. Statistical Analysis

Three primary questions were addressed: (1) MTR differences across structural states; (2) frequency of pathogenic, benign and VUS variants in ordered, disordered, and missing residues; and (3) relationships between structural state, MTR, variant class, and sequence composition/segment patterning in NDD proteins.

MTR distributions across four states (disorder, order, structural transition, missing residues) were summarized using descriptive statistics (skewness and kurtosis) and visualized with violin plots, boxplots, and histograms.

Global differences in MTR across states were assessed using a Kruskal–Wallis test, followed by FDR-corrected Dunn’s post hoc tests for pairwise comparisons.

To identify regions under strong missense constraint, observed missense counts were compared to expected proportions using FDR-adjusted exact binomial tests.

For composition analyses at the aminoacid level, residues were classified as Intolerant (MTR < 0.4) or Tolerant (MTR ≥ 0.4). Aminoacids enrichment was was then assessed using two-sided Fisher’s exact tests with the Haldane–Anscombe correction, and results were reported as log_2_ odds ratios (ORs).

Variant prevalence across structural states was summarized using Wilson 95% confidence intervals and compared using FDR-adjusted two-proportion z-tests. Enrichment or depletion of ClinVar pathogenic, benign, and VUS variants by structural state was evaluated using Fisher’s exact tests (state versus all other states), with Benjamini–Hochberg correction applied to derive q-values.

Segment-level analyses compared continuous Intolerant and Tolerant stretches within each structural state. Differences in mean segment length, aromatic spacing (mean F/Y/W distance), and physicochemical properties (net charge, hydropathy, aromatic fraction) were tested using two-sided Mann–Whitney U tests (*p* < 0.05). Physicochemical contrasts are reported as Δ = Intolerant − Tolerant.

## 3. Results

### 3.1. NDD-Associated Proteins Dataset and Functional Representation

**Figure 2:**
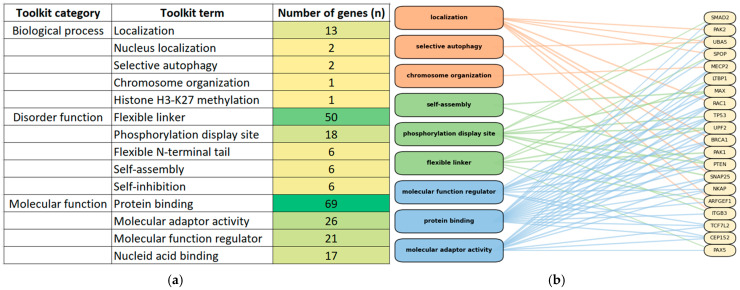
Distribution of GO and IDPO functional terms curated in DisProt for NDD-associated proteins. (**a**) Summary of GO (Biological process and Molecular function) and IDPO terms, showing the number of NDD-associated genes linked to each term annotated in DisProt. Color intensity (green gradient) reflects the number of genes associated with each term, ranging from light to dark green. (**b**) Bipartite network connecting the most representative GO/IDPO terms (**left**) to the subset of NDD-associated genes (**right**). Orange nodes denote GO Biological Process terms, green nodes denote Disorder Function terms, and blue nodes denote GO Molecular Function terms.

We first analyzed the distribution of GO and IDPO curated functional terms annotated in DisProt. Figure 2 provides a descriptive overview of the number of genes associated with each GO and IDPO term. Terms represented by very few genes should be interpreted cautiously, as they provide limited evidence for general functional trends. The disorder state mainly acts as a flexible linker and protein binding (Figure 2a). Several genes (Figure 2b) show the same function, suggesting that DisProt-annotated regions repeatedly fall into a similar functional logic: IDRs that support interaction with other partners, modulation, and context-dependent regulation.

**Figure 3:**
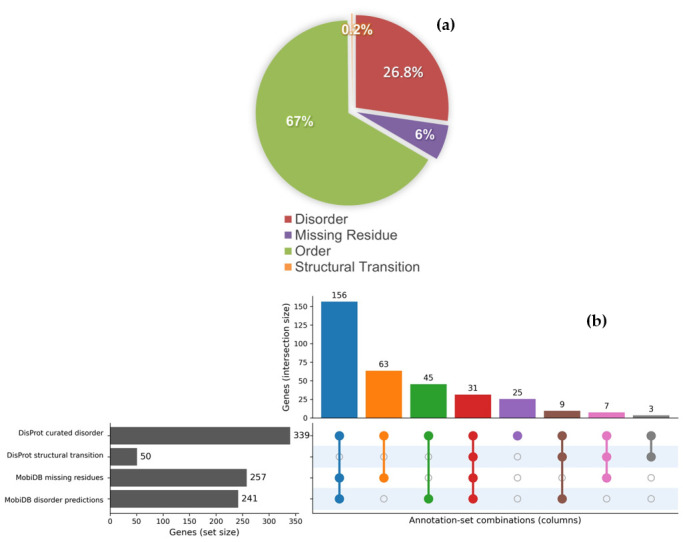
Structural annotation sources and residue state composition in NDDs dataset. (**a**) Distribution of residues across four structural states. (**b**) UpSet plot showing the overlap of annotation types (disorder, structural transition, missing residues), sources (DisProt and MobiDB) and evidence (curated, predictions). Vertical bars indicate the number of proteins in each exact annotation combination, with the dot matrix highlighting which annotations are present (filled) or absent (open). Connecting lines link annotations co-occurring within the same proteins. Horizontal grey bars (**right**) indicate the total number of proteins annotated by each source. Categories are not mutually exclusive, as proteins may carry multiple annotation types.

In order to investigate potential links between MTR score and missense variants implicated in disorder effects, we first characterized the structural composition of the NDD protein dataset. Approximately one third of all residues were annotated as experimentally validated disorder residues, with additional information corresponding to structural transitions or residues missing from experimental structures. Ordered regions remained the largest category, consistent with the presence of structured functional domains embedded within flexible regulatory segments. This heterogeneous distribution reflects the modular architecture typical of NDD-associated proteins, where folded domains coexist with disordered regions (Figure 3a). Comparison of the curated with predicted disorder annotations reveals their complementary contribution to describing disorder-related features in NDD-associated proteins dataset (Figure 3b). Genes lacking any of the four annotations are classified as ordered-only.

### 3.2. Missense Constraint Across All Structural States in NDD-Associated Proteins

We also compared the MTR score to known subregional intolerance across different structural states. Overall, the four structural states show only modest differences in missense tolerance (Table 1): ordered regions were slightly more constrained (mean 0.86) than disordered regions (mean 0.91), while structural transition regions (disorder-to-order) exhibited MTR values comparable to ordered domains (mean 0.88). Despite these global trends, all structural states displayed broad MTR distributions and heavy-tailed behavior, indicating substantial heterogeneity within each category.

**Figure 4:**
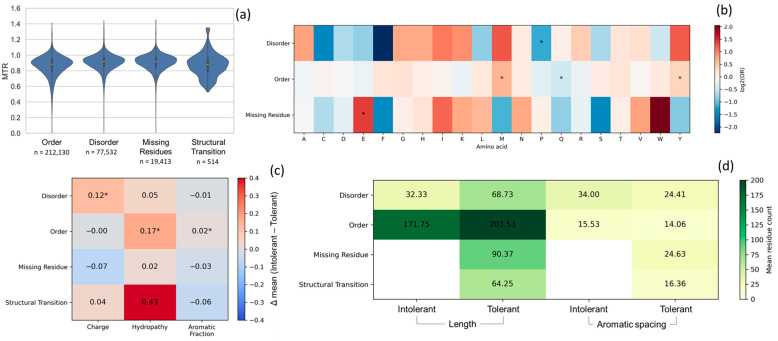
Sequence constraint, composition, and patterning across four structural states (disorder, missing residue, order, and structural transition states) in NDDs dataset. (**a**) Violin plots show residue-level MTR distributions across four structural states. Violin width represents the MTR density, with boxes and dots indicating the IQR and the median respectively. (**b**) Log2(OR) of amino acids enrichment in Intolerant vs. Tolerant across four structural states (*p* < 0.05, two-sided Fisher’s exact tests with Haldane–Anscombe correction). Red (log2OR > 0.0) = Intolerant, blue (log2OR < 0.0) = Tolerant, white ≈ no difference. (**c**) Differences in mean biophysical properties between Intolerant and Tolerant residues within each structural state (Δ = Intolerant − Tolerant) (*p* < 0.05, Mann–Whitney U test). (**d**) Segment-level analysis showing mean segment length and aromatic spacing for Intolerant and Tolerant segments within each structural state. “Length” indicates the mean run length (number of consecutive residues) per segment class; “Aromatic spacing” summarizes the mean distance between aromatic residues (F/Y/W) within segments. MTR threshold: Intolerant (MTR < 0.4), Tolerant (MTR ≥ 0.4); statistical significance is indicated by asterisks (*p* < 0.05). Abbreviations: MTR, Missense Tolerance Ratio; IQR, interquartile range; OR, odds ratio.

This variability is clear in IDRs, where most residues tolerate substitutions but specific subregions remain strongly constrained (StdDev 0.107–0.155) as illustrated in Figure 4a.

To further characterize constraints among states, residues classified as Intolerant (MTR < 0.4) and Tolerant (MTR ≥ 0.4), were compared for composition and biophysical properties. Modest but consistent differences in IDRs, indicating that constrained disordered segments are associated with specific compositional features (Figure 4b–d). Intolerant sites in IDRs show a significant depletion of proline (P), suggesting that selective constraint is concentrated in specific positions rather than uniformly distributed across the sequence. In contrast, ordered regions exhibit minimal compositional differences between Intolerant and Tolerant positions (ΔFrequency ≈ 0), with a significant enrichment of M (Intolerant) and shifts for Q and Y (Figure 4b). Missing residues are enriched for Intolerant E, while structural transition regions are not represented in the residue-level enrichment heatmap due to having no Intolerant positions. Structural transition regions show intermediate constraint levels overall (mean 0.88), but no residues meet our stringent ‘Intolerant’ cutoff (MTR < 0.4). This indicates that transition regions are moderately constrained overall (consistent with their role in regulated binding/folding) without exhibiting the extreme low-MTR tail observed in other contexts.

Physicochemical and segment-level analyses (Figure 4c,d) further support a model in which missense constraint in IDRs is context- and composition-dependent. Intolerant residues in IDRs are enriched in positive charge, whereas segment-level analyses reveal that constrained regions form short, localized stretches are embedded within otherwise tolerant disordered sequences. In contrast, ordered regions form longer contiguous segments with tighter aromatic spacing, missing and structural transition states lack Intolerant segments. Tolerant segments showing intermediate lengths and tighter spacing are more present in structural transition than in missing residues.

### 3.3. Pathogenic Variants Prefer Ordered Regions, While Benign and VUS Variants Accumulate in Disordere Regions

**Figure 5:**
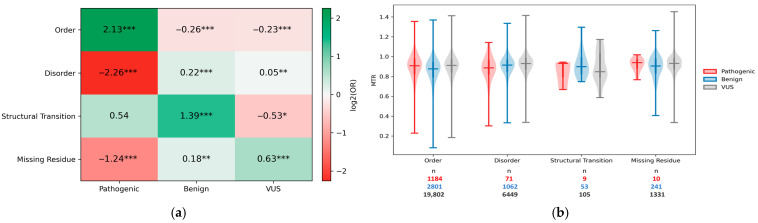
Enrichment analysis of ClinVar missense clinical classes across structural states using Fisher’s exact test. (**a**) Heatmap shows the log2(OR) for enrichment or depletion of ClinVar-annotated missense variants across four structural states. Positive values (green) indicate enrichment and negative values (red) indicate depletion. Each cell is annotated with the corresponding log2(OR) and BH–FDR-adjusted significance (* q < 0.05, ** q < 0.01, *** q < 0.001). (**b**) Violin plot shows the distribution of MTR across four structural states and ClinVar clinical classes for all annotated residues. Violin width reflects the density of observations across the MTR range, while central markers indicate the median and interquartile range for each group.

To assess how variants relate to structural context, we analyzed 33,124 ClinVar variants mapped in 339 NDD-associated proteins and tested for enrichment or depletion of pathogenic, benign, and VUS annotations across structural states using Fisher’s exact tests (each state vs. all other states combined; BH–FDR correction) (Figure 5a). Pathogenic variants are significantly enriched in ordered regions (log2OR = 2.13, q < 0.001) and strongly depleted in IDRs (log2OR = −2.26, q < 0.001) and missing residues (log2OR = −1.24, q < 0.001), while structural transition display a weak, non-significant pathogenic enrichment (Figure 5a). Non-pathogenic classes exhibit complementary trends: benign annotations are modestly depleted in order and enriched in disorder, while VUS are depleted in ordered regions and show smaller but significant shifts in disorder and missing residues. Structural transition regions display a distinctive profile, characterized by strong enrichment for benign variants and depletion for VUS, without a corresponding significant enrichment of pathogenic annotations (Figure 5a).

Together, these results indicate that the distribution of ClinVar missense classes is not uniform across structural states. To relate these annotation biases to constraint, we examined residue-level MTR distributions across both structural state and ClinVar class (Figure 5b). The figure shows that, within each structural state, pathogenic-, benign-, and VUS-labeled residues exhibit broad, overlapping MTR distributions (with similar medians and wide ranges), indicating that clinical labels alone do not separate residues into discrete “tolerant” vs. “intolerant” MTR categories within a given state. This supports a view of constraint as heterogeneous and locally concentrated within each structural state.

### 3.4. Evaluating Missense Variations by Local Constraints in Two Use Cases

**Figure 6:**
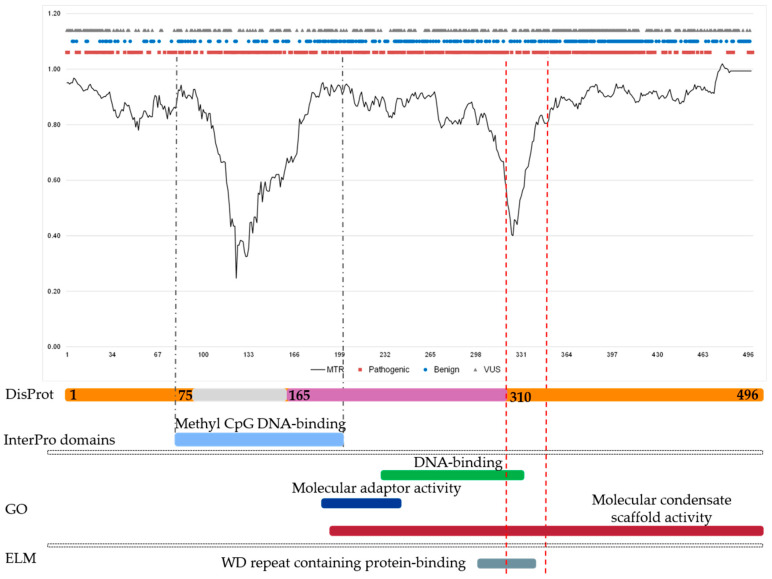
Mecp2 protein involved in NDDs, showing long IDRs and constrained regions. From top to bottom: MTR profile along the Mecp2 amino acid sequence. ClinVar missense variants, grouped by clinical significance, are mapped to their corresponding positions. DisProt annotations indicate disorder (red), disorder-to-order transitions (orange), and ordered regions (grey). Functional domains (InterPro). Curated GO annotations for disordered and disorder-to-order regions are shown. Short linear motifs (ELM), and phase separation-related features (PhasepDB) are annotated below the sequence, providing an integrated view of structure, function, and constraint. Abbreviations: GO, Gene Ontology.

To demonstrate that this approach can be used to complement variant interpretation within IDRs and to reassess selected variants, we examined missense variation across disorder-related structural states, focusing on regions with low MTR scores indicative of strong evolutionary constraint and reduced tolerance to amino acid substitutions. As a case study, we analyzed Methyl CpG Binding Protein 2 (MECP2), a DNA-binding protein that mediates transcriptional repression through interactions with histone deacetylases. Pathogenic variants in *MECP2* cause Rett syndrome (OMIM #312750), one of the most extensively characterized neurodevelopmental disorders. Low-MTR regions are detected within the Methyl-CpG Binding Domain (residues 78–162) and within a C-terminal disordered region (residues 310–369). Notably, despite this IDR exhibiting the lowest MTR values, it harbors fewer reported pathogenic variants than the ordered domain. Nevertheless, this disordered segment overlaps with functionally critical regions, including molecular scaffold activity and the WD repeat-binding region, highlighting that strong evolutionary constraint within IDRs may reflect essential regulatory or interaction functions that are not yet fully captured by current variant annotations (Figure 6).

**Figure 7:**
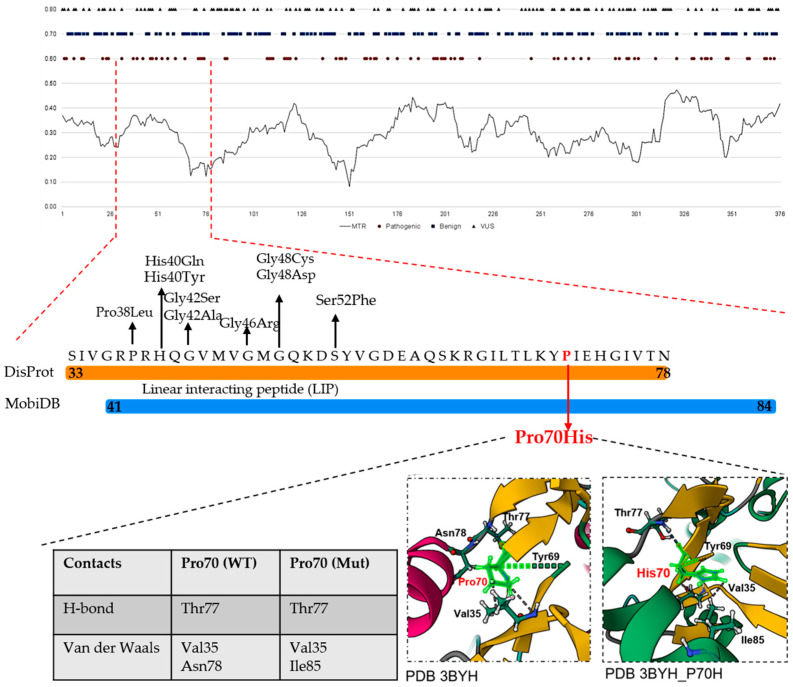
MTR and ClinVar missense variants mapped into the IDR of Actin, cytoplasmic 1 (P60709). From top to bottom, the figure shows the MTR score mapped across the entire protein sequence. The orange box highlights the experimentally validated IDR (residues 33–78), as reported in DisProt, blue box highlights the missing residues as reporetd in MobiDB. Missense variants that are absent from gnomAD and occur at strongly Intolerant residues (MTR < 0.3) are classified as VUS in ClinVar and are displayed along the IDR. The Pro70His VUS, visualized using RING, replaces a van der Waals contact involving Asn70 with a new interaction with Ile85. Although all mapped missense variants have been reported in individuals affected by Baraitser–Winter syndrome 1, none are currently classified as pathogenic.

Figure 7 illustrates how, within the IDR of Actin—cytoplasmic 1 (UniProt: P60709; DisProt: DP03957) corresponding to the lowest MTR values (<0.20), the Pro70His variant, currently classified as VUS, may warrant reevaluation through a more detailed structural analysis. Notably, uncertain variants have been mapped within IDR; these variants are absent from gnomAD and are reported in ClinVar in association with Baraitser–Winter syndrome 1 (BRWS1). In particular, detailed analysis using the RING tool [34] indicates that the Pro70His substitution disrupts the interaction with Asn78 while establishing a new van der Waals contact with Ile85, thereby suggesting a local reorganization of the interaction network with potential structural consequences.

## 4. Discussion

This study set out to quantify how genetic constraint is distributed across ordered and disordered regions in a curated dataset of NDD-related proteins and to relate this landscape to clinical variant interpretation. By integrating structural annotations (disorder, order, disorder transition and missing residues), MTR score, and ClinVar data, we provide a residue-resolved view of missense constraint in proteins enriched for IDRs. A key contribution of this study is demonstrating that a residue-resolved MTR framework can recover potentially pathogenic missense variants that are overlooked by conventional computational methods in IDRs or missing residues.

### 4.1. Constraint Is Only Modestly Shifted by Global Structural Class, but Highly Localized Within Each Class

Although mean MTR values differ across four structural states, these differences are modest overall. Notably, statistical testing grouped ordered and structural transition regions, and disordered and missing residues, underscoring a clear separation between structurally defined and intrinsically flexible segments. This supports previous observations [35], describing the coexistence of both highly constrained and weakly constrained subregions within IDRs. It also supports the idea that intrinsic disorder can be subject to strong purifying selection when flexible segments are involved in specific interactions or extend folded domains [36,37]. In such cases, sequence variation may disrupt function and is therefore selectively disfavored despite the lack of a stable three-dimensional fold.

Missing residues, derived from experimental structures, add further complexity. These segments are unresolved in crystallographic or cryo-EM density and are often assumed to be flexible or poorly structured [38,39]. The generally higher MTR values may indicate reduced functional constraint, but they may also reflect technical and sampling biases, since structurally unresolved residues are often under-represented in variant datasets.

### 4.2. Sequence Features of Constrained Sites Differ by Structural State

To move beyond global distributions, we classified residues into Intolerant and Tolerant categories and examined amino acid composition, physicochemical properties, and segment-level organization across structural states. In IDRs, missense constraint does not reflect the overall disorder-promoting amino acids composition. Instead, constraint is selectively enriched in specific residue types: A, G, and K are overrepresented among Intolerant positions, whereas P and S dominate among Tolerant positions. This indicates that, even within broadly permissive disordered sequences, constraint is concentrated on specific residue types. In contrast, ordered regions show minimal compositional differences between Intolerant and Tolerant residues, consistent with constraint being driven primarily by structural geometry rather than amino acid identity. These findings are consistent with established compositional biases of IDRs, which are enriched in hydrophilic and flexible residues such as S, A, K, E, G, Q, and R, as well as selected hydrophobic residues including M and P [40,41,42]. Structural transition regions, which comprise less than 1% of analyzed positions, are dominated by Tolerant residues [42]. Physicochemical properties reveal subtle state-specific differences rather than biophysical extremes: Intolerant and Tolerant residues across states exhibit near-neutral charge, mild hydrophilicity, and low aromatic content, implicating nuanced recognition motifs. Segment-level analyses further support this view, with ordered regions forming longer contiguous stretches and tighter aromatic spacing, while disordered segments are shorter and more loosely patterned, consistent with models of short functional elements embedded within IDRs [43].

### 4.3. Structural Bias in Clinical Annotation of Missense Variants

Mapping ClinVar variants onto constraint landscapes shows that pathogenic missense variants are enriched in ordered and transition regions, whereas benign variants are more common in disordered and missing segments. This agrees with prior reports of pathogenic variant depletion in IDRs [44,45], and with the observation that folded domains are generally subject to stronger evolutionary and functional constraints. However, these trends must be interpreted cautiously. IDRs are poorly captured by current variant-effect predictors and are often under-annotated functionally [44,45]. As a result, variants in ordered domains are more likely to be studied and classified, whereas variants in IDRs are labeled as VUS or benign due to limited evidence [18,46]. The relatively even distribution of VUS across states in our dataset is consistent with this gap. The subset of residues with very low MTR illustrates this point. Only a small fraction of these strongly constrained sites currently carry benign or pathogenic labels, and nearly all lie in ordered regions. This suggests that many constrained sites in disordered or missing segments remain clinically unexplored. Overall, our results support a model in which missense constraint in IDPs is highly localized and context-dependent, reinforcing emerging motif-centric frameworks for interpreting variation in IDRs [45,47].

This has important implications for variant interpretation. Conventional variant-effect predictors (VEPs) that rely heavily on conservation in multiple sequence alignments or on changes to predicted stability perform poorly in IDRs [44]. Incorporating residue-level, together with IDR functional annotations (e.g., protein binding, flexible linker, molecular adaptor activity, etc.), may help distinguish missense variation that is likely to have functional consequences.

## 5. Conclusions and Limitations

Here we present the MTR analysis to explore regional intolerance to missense variation across 339 human IDPs associated with NDDs. Using MTR calculated with 730,947 exomes and 76,215 genomes from gnomAD v4.1.0, we provide a framework for identifying constrained sites within IDRs and highlight variants of uncertain significance that may have functional or clinical relevance. Our results highlight the importance of explicitly considering the distinct evolutionary and functional properties of IDRs in clinical variant interpretation. The implementation of MTR scores as a complementary metric in diagnostic algorithms may aid in identifying and prioritizing variants in these complex and heterogeneous disorders. Within clinical workflows, including those guided by the American College of Medical Genetics and Genomics (ACMG) guidelines [48], automated classification tools such as InterVar [49] may further support the evaluation of variants located in IDRs. However, MTR is based on population-level variation and is not a stand-alone diagnostic measure. Its clinical applicability is limited by factors such as population sampling biases, the lack of validated thresholds, and the absence of integration with patient-specific phenotypic or segregation data.

These results should also be considered in light of current limitations in dataset size, clinical annotation and variant interpretation, which disproportionately affect disordered regions and may lead to under-recognition of constrained sites outside ordered domains. Given that more than 1500 genes are robustly associated with NDDs, expanding disorder-aware frameworks to a broader set of proteins represents an important future direction. Enrichment analyses reveal a strong structural bias in clinical annotations, and a systematic comparison between MTR-based approaches and established motif-annotation frameworks for IDRs in NDDs is an important avenue for future work.

## Figures and Tables

**Table 1:** Descriptive statistics and pairwise comparisons of Missense Tolerance Ratio (MTR) across structural states in NDD proteins.

Structural Status	*n*	Mean	StdDev	Skewness	Kurtosis	Dunn Group (FDR 0.05)
Order	212,130	0.86	0.14	−1.15	6.59	a
Disorder	77,532	0.91	0.11	−0.58	7.06	b
Missing Residues	19,413	0.91	0.11	−0.95	10.33	b
Structural Transition	514	0.88	0.15	0.52	3.87	a

Groups sharing the same letter are not significantly different (Dunn test, FDR-adjusted *p*, α = 0.05). Groups with different letters differ significantly. Abbreviations: *n*, number of residues; StdDev, standard deviation; FDR, False Discovery Rate.

## Data Availability

The data that support this study are openly available in DisProt at https://disprot.org/ and MobiDB https://mobidb.org/.

## Supplementary Materials

The following supporting information can be downloaded at: https://www.mdpi.com/article/10.3390/genes17020219/s1, Table S1: List of DisProt proteins associated with NDDs and corresponding ClinVar variant counts.
